# Exploring the Phosphoregulatory Network of Human Sucrose Non-Fermenting 1-Related Kinase

**DOI:** 10.3390/biology15090709

**Published:** 2026-04-30

**Authors:** Vaishnavi Gopalakrishnan, Amal Fahma, Athira Perunelly Gopalakrishnan, Suhail Subair, Prathik Basthikoppa Shivamurthy, Rajesh Raju, Sowmya Soman

**Affiliations:** Centre for Integrative Omics Data Science (CIODS), Yenepoya (Deemed to be University), Mangalore 575018, Karnataka, India; gvaishutiruppur@gmail.com (V.G.); amalfahma7@gmail.com (A.F.); 19228@yenepoya.edu.in (A.P.G.); suhailrejeena007@gmail.com (S.S.); prathikbsgowda@gmail.com (P.B.S.); rajeshraju@yenepoya.edu.in (R.R.)

**Keywords:** SNRK, predominant phosphosites, phosphoregulation, coregulation, co-occurrence

## Abstract

Sucrose non-fermenting 1-related kinase (SNRK) is an understudied serine/threonine kinase of the CAMKL family, known for its role in metabolic regulation and cell signaling. Despite its emerging relevance in various biological processes and diseases, the phosphoregulatory landscape of human SNRK (valid substrates or role of its phosphosites) remains unexplored and demands robust, large-scale, data-oriented approaches to predict the potential substrates. A comprehensive analysis of global human phosphoproteomics datasets was performed to systematically identify class I phosphosites on SNRK, along with their predicted upstream kinases, potential downstream substrates, and coregulated phosphoproteins. Our analysis resulted in the identification of 33 dark SNRK phosphosites, of which 19 were differentially regulated across an array of experimental conditions. Among them, S518 and S569, outside their kinase domain, were the most frequently regulated and co-occurred phosphosites under diverse conditions. Notably, S569 is predicted as a candidate autophosphorylation site of SNRK. In these contexts, coregulation analysis of proteins and their phosphorylation sites suggested associations of phospho-SNRK in cell cycle progression, chromatin organization, and DNA replication. Uncovering candidate upstream kinases and potential substrates for prioritized validation, this study provides the first comprehensive phosphoproteomic map of SNRK, serving as a foundation for future investigations into its signaling network associations and therapeutic approaches.

## Simple Summary

SNRK, widely recognized as a stress-responsive kinase in plants but largely unexplored in humans, is emerging as an important regulator of metabolism. It is implicated as a potential therapeutic target in metabolic pathological conditions, including metabolic dysfunction-associated fatty liver disease (MAFLD) and insulin resistance. However, gaps remain in our understanding of its phosphosite-level functional associations, as well as its upstream kinases and downstream substrates. Due to the unavailability of its complete crystal structure, data-driven phosphoproteomics analysis of SNRK was employed. This study found the previously uncharacterized phosphosites S518 and S569 as predominant sites due to recurrent detection across datasets. Their kinase regulatory network and its phosphosite association with other proteins were inferred from the assembled phosphoproteomics datasets.

## 1. Introduction

Sucrose non-fermenting 1-related kinase (SNRK) is a relatively understudied serine/threonine kinase in humans that plays a vital role in regulating metabolic processes in cardiomyocytes and adipocytes [1,2,3]. According to IUPHAR, it is classified within the calcium/calmodulin-dependent protein kinase-like (CAMKL) family, and is the sole member of the SNRK subfamily [4]. Functionally, SNRK shares similarities with the AMP-activated protein kinase (AMPK) family, particularly in its role in regulating cellular energy metabolism [5]. It is primarily localized in the nucleus and is predominantly expressed in hematopoietic progenitor cells and leukemic cell lines. The gene encoding *SNRK* is located on chromosome 3 at position 3p22.1, and the protein comprises 765 amino acids [6]. Structurally, SNRK contains a serine/threonine kinase domain spanning the residues from 16 to 269, a hinge region (residues 271_291), followed by a unique ubiquitin-associated (UBA) domain between residues 292 and 334 [7].

SNRK is primarily involved in regulating hematopoietic cell proliferation and serves as a mediator of neuronal apoptosis [6,8]. In addition to these roles, it has been implicated in several key metabolic signaling pathways, including β-catenin signaling, Rho-associated kinase signaling, Notch, mTOR, and TGF-β pathways [1,9,10,11,12]. Post-translational modifications (PTMs) play a major role in modifying the kinase activity. To date, LKB1 (also known as STK11) is the only kinase reported to phosphorylate SNRK at Thr173 within its kinase domain, thereby activating it [13]. Besides phosphorylation, no other PTMs have been reported in human SNRK. However, it remains unclear whether SNRK functions upstream or downstream of any specific protein phosphosite within these pathways.

Recent reports suggest the protective role of SNRK in the heart and liver, which has been proposed as a potential therapeutic target in pathological conditions such as myocardial infarction, metabolic-associated fatty liver disease (MAFLD), insulin-resistant metabolic disorders, and Kawasaki disease [9,14,15,16]. Additionally, SNRK has been implicated in ovarian cancer metastasis and angiosarcoma, and has been shown to inhibit colon cancer cell proliferation [11,17,18]. In breast cancer, SNRK phosphorylation is markedly higher than its expression level [19]. As a serine/threonine kinase, phosphorylation can regulate its activity in the signaling pathway of these pathological conditions. In this study, we assembled and analyzed large-scale global phosphoproteomics datasets to characterize the phosphoregulatory landscape of SNRK.

## 2. Materials and Methods

### 2.1. Assembly and Analysis of SNRK Global Phosphoproteomics Datasets

The methodology employed in this study follows the established protocols previously developed in our laboratory [20]. The global phosphoproteomic datasets of SNRK were assembled from publicly available research articles indexed in PubMed. These datasets were divided into qualitative profiling datasets and quantitative differential datasets comprising class I phosphosites (defined by a localization probability of ≥75% and an A-score of ≥13). The most frequently detected phosphosites across these datasets were considered as predominant phosphosites. To investigate the mutual associations of phosphorylation sites within SNRK, a co-occurrence analysis was performed, where we particularly investigated the co-differential regulation pattern of phosphosite pairs within SNRK. The phosphosite pair differential coregulation frequency is listed in Table S1.

### 2.2. Prediction of Intrinsically Disordered Regions

Intrinsically disordered regions (IDRs) present in SNRK were predicted using the IUPred3 web server [21], a computational predictor that relies on an energy estimation approach. The full-length protein sequence of SNRK was retrieved from UniProt in FASTA format and submitted to the server. Disorder prediction was performed using the ‘long disorder’ mode. The output disorder scores ranged from 0 (ordered) to 1 (disordered). Residues with scores > 0.5 were classified as disordered, while those with scores < 0.5 were considered ordered.

### 2.3. Coregulation Analysis of SNRK Phosphosites with Phosphoproteins

Coregulation analysis of SNRK phosphosites was performed based on the approach described by Priyanka *et al*. [20]. Quantitative differential datasets were compiled to identify the phosphosites in other proteins (PsOPs) that display either a positive or negative coregulation with the predominant phosphosites in SNRK. For each dataset, expression of differential regulation was categorized as upregulated (U) or downregulated (D). Coregulation patterns between SNRK phosphosites and PsOPs were classified into four categories: UU (both upregulated), DD (both downregulated), UD (SNRK upregulated and PsOP downregulated), and DU (SNRK downregulated and PsOP upregulated).

These phosphosite pairs were further classified into positively coregulated phosphosites (UUDD) based on the ratio ∑(nUU + nDD)/∑(nUD + nDU), representing sites that are upregulated or downregulated together with the SNRK phosphosite. Conversely, negatively coregulated phosphosites (UDDU) were identified based on the ratio ∑(nUD + nDU)/∑(nUU + nDD), indicating an opposite coregulation pattern, in which PsOPs are upregulated when the SNRK phosphosite is downregulated, or vice versa. Positive coregulation (UUDD) represents coregulation in the same direction, whereas negative coregulation (UDDU) indicates coregulation in the opposite direction.

To determine statistically significant patterns of coregulation across the quantitative differential datasets, a one-sided Fisher’s exact test (FET) was performed using a contingency table and a formula as follows:

$$\sum p=\;\frac{\left(a\;+\;b\right)!\left(c\;+\;d\right)!\left(a\;+\;c\right)!\left(b\;+\;d\right)!}{n!}\;\sum_{i}\frac{1}{a_{i}!b_{i}!c_{i}!d_{i}!}$$

For each site pair, their differential regulation in experimental conditions was collapsed into a 2 × 2 contingency table comprising: (a) neither site detected [n_00], (b) only one site detected [n_U0 + n_D0 + n_0U + n_0D], (c) both sites detected with opposite regulation [n_UD + n_DU], and (d) both sites detected with unidirectional regulation [n_UU + n_DD].

Potential biases in cellular phosphoproteomics datasets, including the overrepresentation of multi-temporal datasets and datasets derived using the same stimuli as the experimental condition, were taken into consideration. We have applied four criteria, such as *p*-value, coregulation ratio, distinct PMIDs, and different experimental conditions, to obtain high-confidence coregulated PsOPs. PsOPs with a FET *p*-value < 0.05 in UUDD or UDDU categories were considered statistically significant. A coregulation frequency cut-off of 25% were utilized to ensure consistency in coregulation patterns. Experimental codes were generated to categorize all experimental conditions with the same stimuli into a single experimental code. Then, we calculated the number of PMIDs from which the phosphoproteomics data were derived and calculated a PMID confidence value for positive and negative coregulation. We applied the experimental code confidence and PMID confidence cut-offs of ≥3 to these positive and negative coregulations.

### 2.4. Enumerating the Co-Phosphoregulated Kinases and Interactors of SNRK

Within the high-confidence PsOPs, the upstream kinases, substrates, binary and complex interactors from various sources, were examined. The experimentally validated upstream kinases associated with SNRK phosphosites were sourced from PhosphoSitePlus (retrieved on 22 May 2023) [22], Phospho. ELM 9.0 (retrieved on 24 May 2023) [23], and RegPhos 2.0 (retrieved on 24 May 2023) [24]. Predicted upstream kinases of SNRK phosphosites and their predicted substrates were obtained using NetworKIN (retrieved on 4 January 2023) [25], AKID (retrieved on 24 May 2023) [26], iKiP-DB [27], and from the phosphomotif analysis (threshold of 90%) conducted by Johnson *et al*. (2023) [28].

The experimentally validated interactors and complexes involving SNRK were extracted from HPRD (http://www.hprd.org) [29], BIND version 2.0 [30], BioGRID version 4.4.248 [31], and ConsensusPathDb release 35 (downloaded on 22 May 2023) [32], CORUM (downloaded on 3 March 2023) [33], and RegPhos 2.0 (downloaded on 24 May 2023) [24].

### 2.5. Gene Ontology Analysis and Data Visualization

Gene Ontology (GO) analysis was performed using g:Profiler (https://biit.cs.ut.ee/gprofiler/gost) (accessed on 29 June 2025) [34] to identify enriched biological processes, molecular functions, and cellular components associated with the coregulated proteins. The AlphaFold-predicted structure of SNRK was retrieved from the AlphaFold Protein Structure Database and further edited within the same platform [35]. For data visualization, RawGraph (2.0) (https://doi.org/10.1145/3125571.3125585) (accessed on 29 June 2025) and Cytoscape (3.10.3) [36] were used to represent relationships among the proteins. R/Bioconductor package trackViewer (10.18129/B9.bioc. trackViewer) is used for generating lollipop plots.

## 3. Results

### 3.1. Global Phosphoproteomics Data Assembly of SNRK Phosphosites

**Figure 1:**
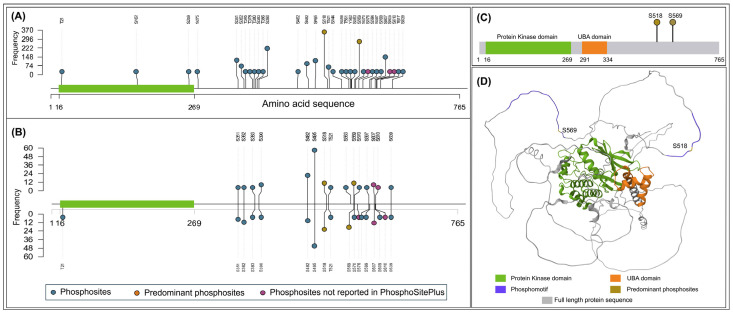
Lollipop plot visualization of the frequency of SNRK phosphosites. Each node represents the phosphosite. (**A**) Class-1 phosphosites were detected in SNRK across our 609 assembled global cellular qualitative profile datasets. (**B**) Class-1 phosphosites were detected in SNRK across our 200 assembled global quantitative differential profile datasets. (**C**) Domain architecture of SNRK. (**D**) AlphaFold-predicted structure, highlighting the functional domain and motifs of predominant phosphosites.

**Figure 2:**
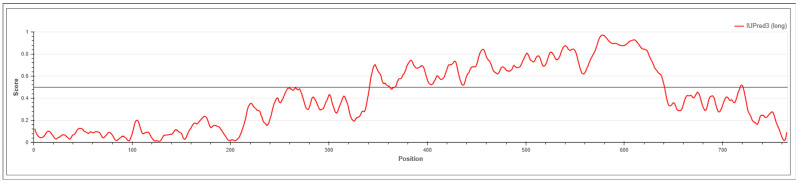
Prediction of intrinsically disordered regions in SNRK using IUPred3. The disorder propensity of SNRK was analyzed using the IUPred3 (long disorder) mode. The x-axis represents amino acid positions, and the y-axis indicates the IUPred3 (long disorder) disorder score ranging from 0 (ordered) to 1 (disordered). The horizontal threshold line at 0.5 distinguishes ordered (<0.5) from disordered (>0.5) regions.

Recent advances in global phosphoproteomics have enabled comprehensive mapping of phosphorylation dynamics across biological systems [37]. A large number of phosphoproteomics datasets are now publicly available in repositories. Towards delineating the phosphoregulatory signaling network of SNRK, a dark kinase whose phosphorylation site functions remain largely uncharacterized, we screened over 3825 publicly available literature containing human phosphoproteomics datasets of SNRK. From the assembled datasets containing class I phosphosites, 609 qualitative profiling datasets and 200 quantitative differential datasets were obtained (Table S2). Systematic mapping led to the identification of 33 phosphosites from profiling data and 19 phosphosites exhibiting differential regulation across various conditions (Figure 1). Among the differentially regulated phosphosites, T21 is located within the kinase domain, while the remaining sites are positioned outside the domain, primarily toward the C-terminal region of the protein. IDRs in SNRK were predicted to identify the structural context of the phosphorylation sites using the IUPred tool [21]. Most of the phosphosites were located in the IDRs (Figure 2). Based on our global phosphoproteomes, S610, S609, and S576 were currently not reported in PhosphoSitePlus [22]. Both S609 and S610 were found to be differentially regulated under metformin treatment [38], and S576 was reported to be downregulated in response to DNA damage [39].

### 3.2. Co-Occurrence of Predominant Sites S518 and S569

**Figure 3:**
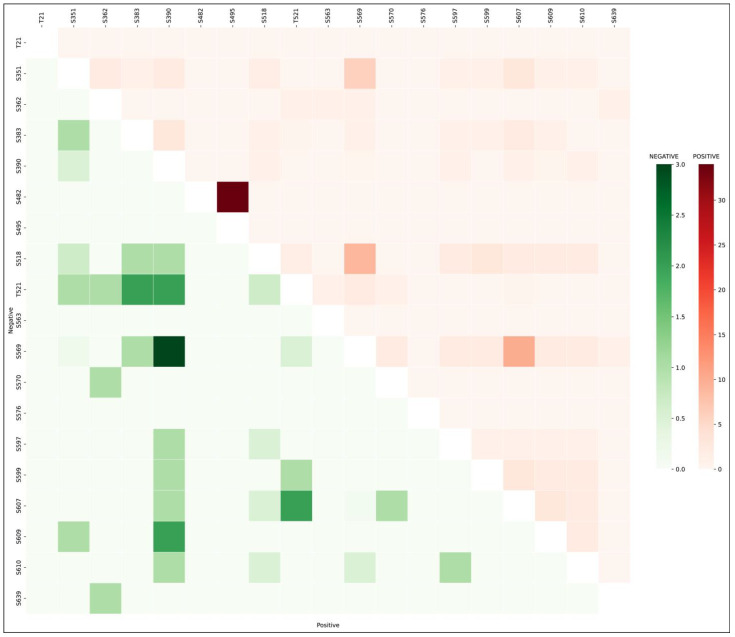
Heat map representing positive and negative co-occurrence patterns of phosphosites within SNRK. Positive co-occurrence with frequency of positive coregulation over negative coregulation is represented in the upper red triangle, and negative co-occurrence with frequency of negative coregulation over positive coregulation is represented in the lower green triangle.

Differentially regulated phosphosites were ranked according to their frequency across datasets to prioritize functionally significant sites. The phosphosite S495, with the highest frequency, appeared mainly in hypoxia-related datasets derived from a single study [40], indicating potential bias due to limited experimental diversity. Therefore, phosphosites S518 and S569 with the next highest frequencies (35 and 32) were selected as predominant sites of SNRK under various conditions. Since differentially regulated phosphosites are in proximity, the co-occurrence of their phosphorylation was examined across the datasets. Phosphorylation sites that co-occur are often regulated under similar biological conditions and may be functionally interconnected [41]. The phosphosites S518 and S569, as well as S569 and S607, exhibited positive co-occurrence (Figure 3). Both predominant phosphosites S518 and S569 were positively co-occurred (either upregulated together or downregulated) in nine datasets. We can also attribute this difference to the existence of multiple prominent phosphoforms of SNRK that may co-exist (S518 phosphoform and S569 phosphoform). S569 and S607 were positively co-occurred in 10 datasets and negatively co-occurred (one is upregulated while the other is downregulated and vice versa) in one dataset. Additionally, according to the cProSite database, SNRK_S518 exhibits elevated expression in breast cancer, while SNRK_S569 shows high expression in ovarian cancer, suggesting potential cancer-type-specific regulatory roles for these phosphosites [42].

### 3.3. Coregulated Phosphoproteins with SNRK

**Figure 4:**
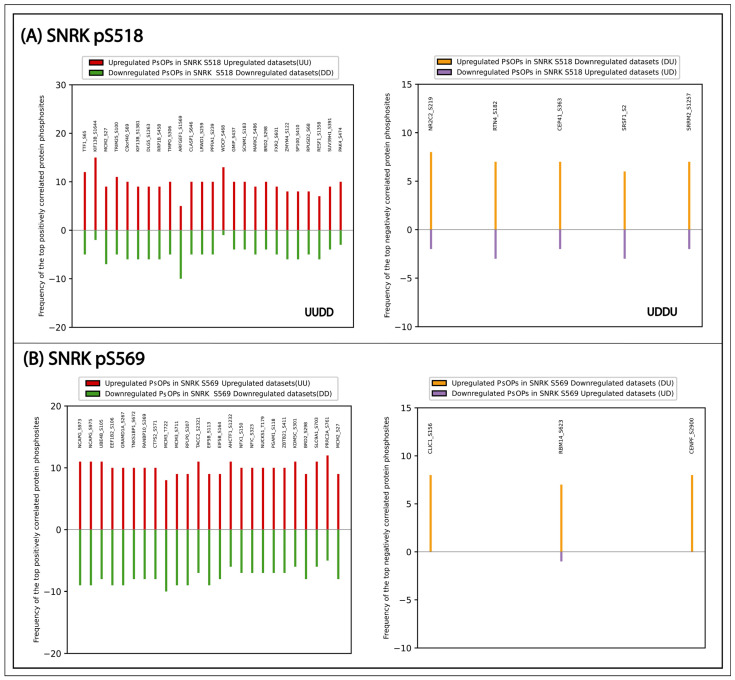
Highly confident, positively and negatively correlated PsOPs of predominant SNRK phosphosites.

To explore the potential functional role of SNRK phosphorylation, co-occurring phosphorylation events in other proteins were examined. Since phosphorylation events that co-occur are often regulated within shared biological processes, a set of coregulated phosphoproteins was identified. They were categorized based on positive and negative coregulation and subsequently applied certain cut-offs to filter high-confidence coregulated phosphorylation in other proteins (PsOPs) (Table S3). As a result, 299 PsOPs showed positive coregulation with SNRK_S518, while 909 PsOPs exhibited positive coregulation with SNRK_S569. In contrast, five PsOPs (NR2C2_S219, RTN4_S182, CEP41_S363, SRSF1_S2, SRRM2_S1257) showed negative coregulation with SNRK_S518, and only three PsOPs (CLIC1_S156, RBM14_S623, CENPF_S2900) were negatively coregulated with SNRK_S569. Among them, the highly confident positively coregulated proteins are given in Figure 4. These top positively coregulated PSOPs associated with SNRK_S518 included TTF1, KIF13B, and MCM2, associated with transcription termination, cortical cytoskeleton, and DNA replication [43,44,45]. Likewise, for S569, proteins such as NCAPG, UBE4B, and MCM3 are involved in chromatin condensation, apoptosis, and DNA replication [46,47,48]. Gene ontology analysis of the positively coregulated proteins of SNRK_S518 revealed enrichment in biological processes such as cell cycle regulation, chromatin organization, and small GTPase-mediated signal transduction. Similarly, for SNRK_S569, enriched biological processes include DNA damage response, transcription regulation, cytoskeleton organization, and cell cycle process (Table S4). Consistent with the observed positive co-occurrence between SNRK_S518 and SNRK_S569, 70 PsOPs exhibited positive coregulation with both phosphorylation sites. Out of these, 13 proteins such as ANKRD17, CDKN1A, CIT, CLASP1, DIS3L2, KNL1, MCM2, NCAPG, RB1, SETD2, TACC2, TACC3 and TPR are related to mitotic cell cycle process.

### 3.4. Phosphosite Coregulation of SNRK Interactors

Protein–protein interactions can be dynamically regulated by PTMs, including phosphorylation [49,50]. The phosphosite coregulation of known interactors of SNRK was also examined. We compiled a list of 34 binary interactors and 29 complex-forming partners from multiple interaction databases (Table S5). Remarkably, among the binary interactors, only plakophilin-3 (PKP3) at S238 and among the complex members, only the casein kinase II β-subunit (CSNK2B) at S209 met our coregulation threshold in SNRK_S518 positive coregulation. CSK2B participates in the Wnt signaling pathway, and its S209 site is a known CDK1 substrate during mitosis [51]. In SNRK_S569 positive coregulation, four binary interactors, including STK11_S31, JUN_S243, PLEKHA4_S194, YTHDC1_S320, and S318, were present. Notably, JUN_S243 is a phosphorylation site known to inhibit its transcriptional activity by reducing DNA binding affinity and is a target of GSK3B [52]. Interactors enriched in this study are based on phosphosite-level coregulation in differential phosphoproteomes and do not infer protein-level interaction.

### 3.5. Kinase Predictions for Predominant SNRK Phosphosites S518 and S569

**Figure 5:**
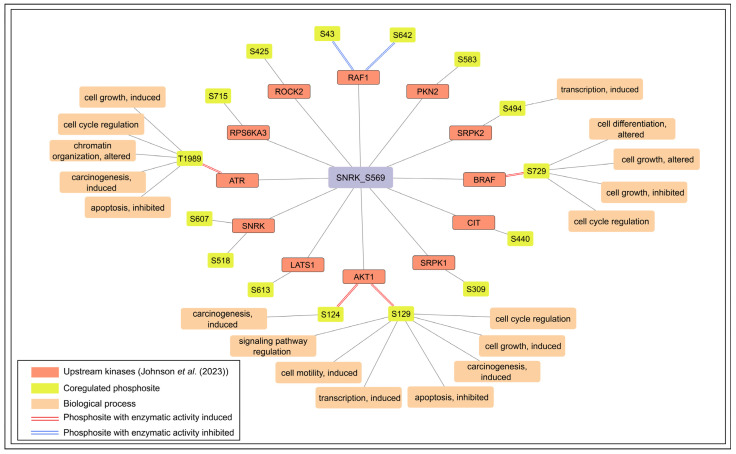
The network depicts the high-confidence expression coregulated phosphosites in upstream kinases that are predicted by Johnson *et al*. (2023) [28].

To understand the signaling mechanism regulating SNRK phosphorylation, it is crucial to identify the upstream kinases responsible for these phosphorylation events. Currently, the kinases responsible for phosphorylating the two predominant phosphosites remain unidentified. However, Johnson *et al*. [28] predicted 10 and 114 potential upstream kinases for S518 and S569, respectively (Table S6). Among them, phosphosites of 16 predicted upstream kinases were positively coregulated with S569 under various experimental conditions (Figure 5). Phosphosites such as AKT1_S124 and S129, ATR_T1989, and BRAF_S729 are known to induce enzymatic activity. Supporting this, seven AKT1 substrates (BABAM1_S29, EDC3_S161, FLII_S436, LARP6_S451, MVD_S96, FOXO1_S256, and FOXO3_ S253) and five ATR substrates (ATR_S74, DCK_S294, PHF14_S12, SUN2_S12, and USP20_S132) were found to be positively coregulated with SNRK_S569. In particular, the coregulated phosphosite SRPK2 S494 was predicted as both an upstream kinase and a downstream substrate of SNRK by Johnson *et al*. [28]. SRPK2_S494 is phosphorylated by RPS6KB1, a kinase downstream of the mTOR signaling pathway [53]. Two phosphosites on RAF1, S43 and S642, known to inhibit its enzymatic activity [54], were found to be positively coregulated with SNRK_S569. AKT1_S129, BRAF_S729, and ATR_T1989 phosphorylation were associated with cell cycle regulation [55,56,57]. Notably, SNRK_S569 showed positive coregulation with SNRK_S518 and SNRK_S607, and Johnson *et al*. [28] also predicted SNRK as an upstream kinase for these sites. These observations suggest that S569 may represent a candidate autophosphorylation site, which requires experimental validation.

### 3.6. Predicted and Coregulated Downstream Targets of SNRK

**Figure 6:**
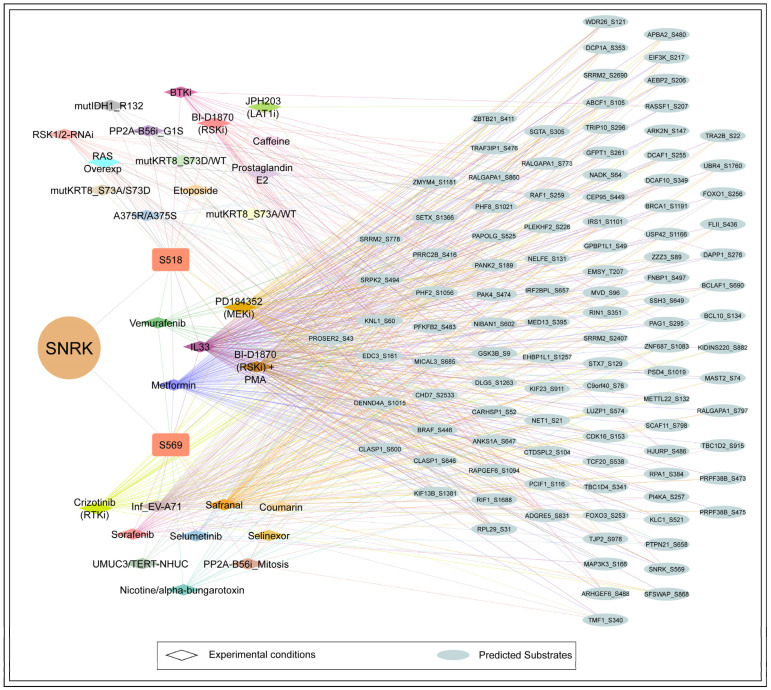
Network visualization of predicted substrates coregulated with SNRK phosphosites S518 and S569 across multiple experimental conditions. Each condition is represented by a distinct color, and edges connecting substrates are color-coded to correspond to the specific conditions under which coregulation was observed. Substrates are arranged in columns based on decreasing frequency of coregulation across conditions, ranging from 10 to 3 conditions from left to right.

From the available databases and the current literature, no experimentally validated substrates for SNRK were identified. Therefore, we next examined predicted substrates that coregulated with SNRK_S518 and S569. Among the 4356 proteins predicted as potential SNRK substrates by Johnson *et al*., 35 and 79 downstream substrates showed positive coregulation with SNRK phosphosites S518 and S569, respectively (Figure 6) (Table S7). These coregulated substrates can serve as potential candidates for experimental validation. SNRK_S569 is associated with a greater number of positively coregulated predicted substrates (79) compared to S518 (35). Phosphosites in seven predicted substrates, including ANKS1A_S647, CLASP1_S600, DENND4A_S1015, DLG5_S1263, KNL1_S62, PRRRC2B_S416, and ZMYM4_S1181, exhibited positive coregulation with both predominant SNRK phosphosites. This limited overlap suggests that each predominant phosphosite is coregulated with several unique substrates. It raises the possibility that phosphorylation at S518 and S569 may impact the substrate selectivity of SNRK, which remains to be experimentally validated. Three coregulated phosphosites (S2690, S778, S2407) of pre-mRNA splicing protein SRRM2 were predicted substrates. Predicted substrates such as CLASP1_S646, KIF13B_S1381, DLG5_S1263, and PAK4_S474 ranked within the top 25 proteins positively coregulated with SNRK_S518, while ZBTB21_S411 was highly coregulated with S569. Of the predicted substrates coregulated with SNRK at S569, phosphosites such as NADK_S64, MVD_S96, BCL10_S134, FOXO3_S253, and GFPT1_S261 are associated with activity-inducing. Similarly, for S518, activity-inducing phosphosites include PAK4_S474, ARHGEF6_S488, GSK3B_S9, RAF1_S259, and BRAF_S446. Substrates coregulated with SNRK at S518, such as BCLAF1, BCL10, FOXO3, and FOXO1, and those associated with S569, including GSK3B, BRCA1, PAK4, and ARHGEF4, are involved in the regulation of apoptosis. We examined individual experimental conditions and observed variability in the coregulation of predicted substrates across different contexts (Figure 6). Notably, 32 and 59 substrates were coregulated with SNRK S518 and S569, respectively, under metformin signaling in colorectal cancer cells [38]. Similarly, 29 and 70 substrates were coregulated with S518 and S569 during interleukin-33 signaling in THP1 monocytes [58]. This observation highlights that the regulatory landscape of SNRK is dynamic and influenced by cellular context, stimulus, and disease state.

## 4. Discussion

SNRK has been increasingly implicated in a variety of physiological and pathological processes, including inflammation, vascular development, angiogenesis, mitochondrial bioenergetics, fatty acid oxidation, and insulin signaling [59]. Analysis of large-scale global phosphoproteomics datasets identified SNRK_S518 and SNRK_S569 as the predominant phosphorylation sites. They are located in IDRs, which highlight their structural flexibility and context-dependent regulatory behavior [60]. However, the functional impact of these phosphosites remains unknown, classifying them as ‘dark phosphosites’. Notably, these phosphosites are located outside the kinase domain and the conserved UBA domain, suggesting that they may play regulatory roles independent of the core catalytic or ubiquitin-associated functions of SNRK. Notably, these sites co-occurred across different experimental phosphoproteomic datasets and exhibited coregulation with a shared set of phosphoproteins involved in cell cycle regulation.

To explore potential functional analogs, we performed a BLAST+ 2.17.0 search using the full-length SNRK sequence, which revealed that SIK1 (Salt-Inducible Kinase 1), also known as SNF1-like kinase (SNF1LK), exhibits the highest sequence similarity (51%). Both SNRK and SIK also possess a conserved UBA (ubiquitin-associated) domain. Pairwise sequence alignment of the kinase domains showed 69.7% similarity between SNRK and SIK. Importantly, SIK is known to regulate the cell cycle [61]. Their sequence homology suggests that SNRK may be associated with cell cycle regulation, which is hypothesis-driven and requires experimental validation. Further, our phosphoproteomics-based coregulation analysis revealed that a significant proportion of SNRK-associated phosphoproteins are involved in mitotic regulation, chromatin condensation, and DNA replication. Functional studies using the kinase-dead SNRK mutant (T173A) in colon cancer cells showed that SNRK suppresses proliferation and tumorigenicity in a kinase-dependent manner by upregulating CacyBP and downregulating β-catenin [11].

Upstream kinases for SNRK_S569 predicted by Johnson *et al*., such as AKT1, BRAF, RAF1, and RPS6KA3, were involved in the mTOR signaling pathway. Recent findings report that SNRK interacts with the raptor component of mTORC1, thereby promoting fatty acid oxidation in the liver through activation of autophagy [9]. Our analysis revealed positive coregulation between SNRK and AKT1, including two enzymatic activity-inducing phosphosites and six coregulated AKT1 substrates. In addition, AKT1 was also predicted as an upstream kinase of SNRK, collectively suggesting phosphorylation-related cross-talk between SNRK and AKT1 signaling pathways. Supporting this, previous studies have shown that SNRK regulates insulin signaling by modulating PPP2R5D phosphorylation, which in turn affects PP2A activity and subsequently influences AKT phosphorylation in both white and brown adipose tissues [15]. Further, in the study of the kinase-dead mutant of SNRK, downregulated proteins such as AKT1_S124, CCNH_T315, CHMP3_S200, DTL_T516, PAGR1_S237, PCNP_S119, RIF1_S1699, STRN_S245, SUZ12_S546, and TOP2A_S1525 in phosphoproteomic analysis of condition empty vector versus knockdown in H9c2 cells showed positive coregulation with SNRK_S569 [62]. Even though they are not predicted as substrates of SNRK, they could be associated in a phosphoregulatory network.

The coregulation of predicted downstream substrates across different conditions showed that SNRK’s regulatory landscape is dynamic and could vary by cell type, stimulus, and disease state. Taken together, our findings uncover a complex, phosphoregulatory network oriented to SNRK, anchored by predominant phosphosites S518 and S569. The identification of predicted upstream kinases and candidate downstream substrates provides a framework for further experimental validation. Structural analysis of SNRK is proposed to provide deeper insights into its interaction specificity.

## 5. Limitations and Future Directions

The phosphoregulation network of SNRK identified in this study is based on the coregulation patterns observed from global phosphoproteomic datasets available for the human SNRK protein. The observed findings are data-driven, and certain limitations exist as the datasets were obtained under different experimental conditions, cell types, mass spectrometry types, and enrichment methods. Transient phosphorylation events and phosphorylation of low-abundance proteins (neuronal proteins or membrane-associated proteins) are often underrepresented in phosphoproteomic datasets. Further, mass spectrometry-based phosphoproteomics has inherent technical limitations that can affect phosphosite detection, such as variability in ionization efficiency, enrichment strategies, and instrument sensitivity.

The predominant phosphosites and their association with specific biological processes inferred from coregulated proteins require experimental validation. Future studies should therefore incorporate targeted experimental approaches such as site-directed mutagenesis and phospho-specific antibodies of predominant phosphosites to assess their functional relevance. In vitro kinase assays can be performed to validate predicted upstream kinases and downstream substrates. Techniques such as proximity labeling, yeast two-hybrid (Y2H), and phosphopeptide pulldown assay can detect phosphorylation-dependent protein–protein interactions. Additionally, other PTMs can be identified by mass spectrometry-based techniques, coupled with appropriate enrichment strategies. However, this analysis provides the direction for targeted phosphorylation site-specific investigations in future research.

## 6. Conclusions

Our study systematically characterizes the phosphoregulation of SNRK, identifying S518 and S569 as predominant sites that may integrate signaling cues across multiple biological processes. These sites are classified as dark phosphosites with currently unknown functional roles. Coregulation analysis, along with predictions by Johnson *et al*., suggests that S569 may represent a potential autophosphorylation site of SNRK. While no direct substrates have been experimentally validated, it poses a significant challenge to the scientific community to prioritize a set of substrates, and hence, our filtered list of coregulated candidates provides a valuable starting point for future validation. Overall, this study provides a resource for exploring SNRK and offers a framework for future experimental investigation of its phosphosite-level associations.

## Data Availability

The original contributions presented in this study are included in the article/Supplementary Material. Further inquiries can be directed to the corresponding author.

## Supplementary Materials

The following supporting information can be downloaded at: https://www.mdpi.com/article/10.3390/biology15090709/s1, Supplementary Table S1: Co-occurrence data of SNRK phosphosites; Supplementary Table S2: Phosphoproteomics Profiling and Differential datasets for SNRK phosphosites; Supplementary Table S3: High-confidence PsOPs coregulated with SNRK expression [after applying filtering parameters]; Supplementary Table S4: Gene ontology analysis of coregulated PsOPs of SNRK phosphosites S518 and S569; Supplementary Table S5: Coregulated PsOPs in binary and complex interactors of SNRK; Supplementary Table S6: Coregulated PsOPs in upstream kinases of SNRK; Supplementary Table S7: Coregulated PsOPs in downstream substrates of SNRK.
